# Characterization and Potential Use of Cuttlefish Skin Gelatin Hydrolysates Prepared by Different Microbial Proteases

**DOI:** 10.1155/2014/461728

**Published:** 2014-06-15

**Authors:** Mourad Jridi, Imen Lassoued, Rim Nasri, Mohamed Ali Ayadi, Moncef Nasri, Nabil Souissi

**Affiliations:** ^1^Laboratoire de Génie Enzymatique et de Microbiologie, Ecole Nationale d'Ingénieurs de Sfax, Université de Sfax, B.P. 1173, 3038 Sfax, Tunisia; ^2^Laboratoire d'Analyse Alimentaire, Ecole Nationale d'Ingénieurs de Sfax, Université de Sfax, B.P. 1173, 3038 Sfax, Tunisia; ^3^Laboratoire de Biodiversité et de Biotechnologie Marine, Centre de Sfax, Institut National des Sciences et Technologies de la Mer, B.P. 1037, 3018 Sfax, Tunisia

## Abstract

Composition, functional properties, and *in vitro* antioxidant activities of gelatin hydrolysates prepared from cuttlefish skin were investigated. Cuttlefish skin gelatin hydrolysates (CSGHs) were obtained by treatment with crude enzyme preparations from *Bacillus licheniformis* NH1, *Bacillus mojavensis* A21, *Bacillus subtilis* A26, and commercial alcalase. All CSGHs had high protein contents, 74.3–78.3%, and showed excellent solubility (over 90%). CSGH obtained by alcalase demonstrated high antioxidant activities monitored by *β*-carotene bleaching, DPPH radical scavenging, lipid peroxidation inhibition, and reducing power activity. Its antioxidant activity remained stable or increased in a wide range of pH (1–9), during heating treatment (100°C for 240 min) and after gastrointestinal digestion simulation. In addition, alcalase-CSGH was incorporated into turkey meat sausage to determine its effect on lipid oxidation during 35 days of storage period. At 0.5 mg/g, alcalase-CSGH delayed lipid oxidation monitored by TBARS and conjugated diene up to 10 days compared to vitamin C. The results reveal that CSGHs could be used as food additives possessing both antioxidant activity and functional properties.

## 1. Introduction

Gelatin is a protein obtained from collagen by heat denaturation. Most commercial gelatins are derived from the skins, hides, and bones of bovine and porcine [1]. Fish skin might be an alternative raw material for gelatin production, because of bovine problems and religions that prohibit the use of porcine. Recently, skin gelatin from various fish species such as grey triggerfish (*Balistes capriscus*) [2], unicorn leatherjacket (*Aluterus monoceros*) [1], brownbanded bamboo shark (*Chiloscylium puntacum*) and blacktip shark (*Carcharhinus limbatus*) [3], and cuttlefish (*Sepia officinalis*) [4] has been extracted and characterized. Gelatin is widely used in food, pharmaceutical, cosmetic, and photographic applications because of its unique chemical and physical characteristics [2]. Furthermore, gelatin may also be used to produce biologically active peptides by protease hydrolysis. Many studies have demonstrated that the enzymatic hydrolysis of proteins improved their functional properties, including solubility, emulsification, and foaming ability, and their biological activities [5]. In this context, fish gelatin hydrolysates having antihypertensive and antioxidant activities have been reported [6].

On the other hand, one of the major problems in food factories is the lipid oxidation which causes food quality deterioration and shortening of shelf life. This unwanted process not only produces offensive odors and flavors but also decreases the nutritional quality and safety of food by forming secondary reaction products, which could reduce the shelf life of food products [7]. Furthermore, consuming oxidative foods is thought to cause serious diseases (heart disease, cancer, stroke, and diabetes). To prevent oxidative deterioration of foods and to provide protection against serious diseases, it is important to inhibit lipids oxidation and free radicals formation occurring in the food stuff and living body. Antioxidants are used to preserve food products by retarding discoloration and deterioration as a result of oxidation. Synthetic antioxidants such as BHT (butylated hydroxytoluene) and BHA (butylated hydroxyanisole) have a strong antioxidant activity, but their use was restricted because of their potential health hazards. Therefore, there is a growing interest to study antioxidative properties in natural sources including some dietary protein compounds.

Bioactive peptides from collagen and gelatin with antioxidant properties have become a topic of great interest for health food and processing/preservation industries [3, 8]. Further, gelatin hydrolysates from brownstripe red snapper (*Lutjanus vita*) skin [9] and from giant squid (*Dosidicus gigas*) tunics [10] have been reported to exhibit antioxidant activity.

Those works become easier, especially with the recent development of* in vitro* methods for simulating the human digestive tract since they are rapid and safe and do not have the same ethical restrictions as* in vivo* methods [11].

The aim of this investigation was to produce cuttlefish-skin gelatin hydrolysates with different DHs obtained by using several proteases and to study their compositions, antioxidant activities and stability, and water and oil-holding properties. In addition, to test the antioxidant stability of hydrolysates, pH and thermal treatments were also investigated. Then,* in vitro* digestion model system was used to simulate the process of human gastrointestinal digestion (HGID) and to evaluate antioxidant stability using various tests. Furthermore, gelatin hydrolysate showing the strongest antioxidant activities was selected for antioxidant assessment against lipid deterioration in cooked turkey meat sausage during 35 days of storage.

## 2. Materials and Methods

### 2.1. Materials

1,1-Diphenyl-2-picrylhydrazyl (DPPH), bile salt, butylated hydroxyanisole (BHA), *β*-carotene, *α*-tocopherol, glycine, ammonium sulphate, and linoleic acid were purchased from Sigma Chemical Co. (St. Louis, MO, USA). Pepsin was purchased from MP Biomedicals (France). Thiobarbituric acid (TBA) was purchased from Suvchem (MH, India). Modified starch (E1422) was provided from Sigma Chemical CO., St Louis, MO. Potassium ferricyanide, trichloroacetic acid (TCA), ferrous chloride, ferrozine, sodium hydroxide, Tween 40, NaCl, NaNO_2_, and tripolyphosphate (TPP) were of analytical grade.

### 2.2. Cuttlefish Skin Preparation

Cuttlefish by-products were obtained from marine processing industry “IMPEX” located in Sfax city, Tunisia. The samples were packed in polyethylene bags, placed in ice with a sample/ice ratio of approximately 1 : 3 (w/w). They were washed twice with water to eliminate the dark ink, which consists of a suspension of melanin granules in a viscous colorless medium. Finally, cuttlefish outer skin was collected and then stored in sealed plastic bags at −20°C until used for gelatin extraction and analysis.

### 2.3. Enzyme Preparation

Alcalase 2.4 L serine-protease from* Bacillus licheniformis* was supplied by Novozymes (Bagsvaerd, Denmark). Crude enzyme preparations from* Bacillus licheniformis* NH1 [12],* Bacillus mojavensis* A21 [13], and* Bacillus subtilis* [14] were prepared in our laboratory. To measure alkaline protease activity, one unit of protease activity was defined as the amount of enzyme required to liberate 1 *μ*g of tyrosine per minute under the experimental conditions used.

### 2.4. Gelatin Extraction

In order to remove noncollagenous proteins, washed skins were first soaked in 0.05 M NaOH with a skin/solution ratio of 1/10 (w/v) for 2 h at 4°C and the solution was changed every 30 min. The alkaline-treated skins were then washed with cold tap water until neutral pH wash water was obtained. The alkaline-treated skins cuttlefish were soaked in 0.1 M acetic acid with a solid/solvent ratio of 1 : 10 (w/v) and subjected to hydrolysis with pepsine at 15 units/g alkaline-treated skin as described by Jridi et al. [4]. The mixtures were stirred for 48 h at 4°C. To inactivate enzymes, the pH of the mixture was then raised to 7.5 using 10 M NaOH and stirred gently for 1 h at 4°C. Enzymatic-treated skin mixture was then incubated at 40°C for 18 h with continuous stirring to extract the gelatin from the skin.

The mixtures were centrifuged at 10000 g for 30 min using a refrigerated centrifuge to remove insoluble material. The supernatant was collected and freeze-dried (Bioblock Scientific Christ ALPHA 1-2, IllKirch-Cedex, France). The powder obtained referred to as cuttlefish-skin gelatin (CSG) was stored at 4°C until used.

### 2.5. Production of Gelatin Hydrolysates

The skin gelatin was dissolved in distilled water (1%; w/v) and subjected to enzymatic hydrolysis for 3 h under optimal temperature and pH conditions with an enzyme/substrate ratio of 30/1 (U/mg). The optimal conditions were as follows: alcalase (pH 8.0, 50°C), A21 proteases (pH 8.5, 50°C), A26 proteases (pH 8.0, 45°C), and NH1 proteases (pH 10.0, 50°C). The gelatin solutions were allowed to equilibrate for 30 min before hydrolyses were initiated. Enzymes were used at the same activity levels to compare hydrolytic efficiencies. During the reaction, the pH of the mixture was maintained constant by continuous addition of NaOH 4 N. To inactivate enzymes, the solution was heated for 20 min at 80°C.

Finally, the solutions were then centrifuged at 5000 g for 20 min and soluble fractions were freeze-dried and stored at −20°C for further use. The degree of hydrolysis (DH), defined as the percent ratio of the number of peptide bonds broken to the total number of peptide bonds in the protein substrate, was determined according to Adler-Nissen [15].

### 2.6. Chemical Analysis

The moisture, ash, and fat contents of CSG and CSGHs powder were determined according to the AOAC methods [16]. The protein content was determined by Kjeldahl method according to the AOAC method. A factor of 5.5 was used to convert the nitrogen value to protein [16]. All measurements were performed in triplicate.

Analyses of calcium (Ca^2+^), magnesium (Mg^2+^), sodium (Na^+^), potassium (K^+^), chlorure (Cl^−^), nitrate (NO_3_^−^), and sulphate (SO_4_^2−^) contents in freeze-dried hydrolysates were carried out using the inductively coupled plasma optical emission spectrophotometer (ICP-OES) (model 4300 DV, Perkin Elmer, Shelton, CT, USA) according to the method of AOAC [16]. Sample (1 g) was mixed with 1 mL of 70% (v/v) nitric acid. The mixture was heated on the hot plate until digestion was completed. The digested sample was transferred to a volumetric flask and the volume was made up to 10 mL with deionized water. The solution was then subjected to analysis.

In order to determine the amino acid composition, CSG and CSGHs were dissolved in distilled water at 1 mg/mL and 50 *μ*L of each sample was dried and hydrolysed in vacuum-sealed glass tube at 110°C for 24 h in the presence of constant boiling 6 N HCl containing 1% (w/v) phenol and using norleucine as internal standard. After hydrolysis, samples were again vacuum-dried, dissolved in application buffer, and injected into a Beckman 6300 amino acid analyzer (Beckman Instruments Inc., Fullerton, California, USA).

### 2.7. Functional Properties of CSGHs

#### 2.7.1. Solubility

Solubility of CSG and CSGHs was carried out over a wide range of pH values from 2.0 to 11.0 as described by Tsumura et al. [17], with slight modifications. Briefly, 200 mg of freeze-dried hydrolysates of cuttlefish gelatin was suspended in 20 mL deionized distilled water and the pH of the mixture was adjusted to different values using either 2 N HCl or 2 N NaOH solutions. The mixtures were stirred for 10 min at room temperature (25 ± 1°C) and then centrifuged at 8000 g for 10 min. After appropriate dilution, the nitrogen content in the supernatant was determined by Biuret method. The nitrogen solubility of the CSGHs, defined as the amount of soluble nitrogen from the total nitrogen, was calculated as follows:

(1) Nitrogen  solubility(%) =Supernatant  nitrogen  concentrationSample  nitrogen  concentration×100.

#### 2.7.2. Emulsifying Properties

The emulsifying activity index (EAI) and the emulsion stability index (ESI) of the CSG and CSGHs were determined according to the method of Pearce and Kinsella [18] with a slight modification. Gelatin hydrolysate solutions were prepared by mixing freeze-dried CSGHs in distilled water (pH = 7) for 30 min at 60°C with different concentrations (0.1, 0.5, and 1% (w/v)). Thirty milliliters of each CSGH solutions was homogenized with 10 mL of soybean oil for 1 min at room temperature (25 ± 1°C) using Moulinex R62 homogenizer. Aliquots of the emulsion (50 *μ*L) were pipetted from the bottom of the container at 0 and 10 min after homogenization and diluted 100-fold with 0.1% SDS solution. The absorbance of the diluted solutions was measured at 500 nm. The absorbances measured immediately (*A*_0_) and 10 min (*A*_10_) after emulsion formation were used to calculate the emulsifying activity index (EAI) and the emulsion stability index (ESI). All determinations are means of at least three measurements. Consider

(2) $$\begin{array}{c} \text{EAI}\left({\text{m}}^{2}/\text{g}\right)=\frac{2\times 2.303\times A_{0}\times N}{\varphi \times C\times 10,000}, \end{array}$$

where *N* represents a dilution factor, *C* is the weight of protein per unit volume (g/mL), and *φ* is the oil volumetric fraction (0.25).

ESI represents the difference of EAI at 0 and 10 min at 500 nm and was calculated using the next formula:

(3) $$\begin{array}{c} \text{ESI}\left(\min\right)=\frac{A_{0}\times \Delta T}{\Delta A}. \end{array}$$

#### 2.7.3. Foaming Properties

Foam expansion (FE) and foam stability (FS) of CSGHs were determined according to the method of Shahidi et al. [19], with a slight modification. Twenty milliliters (*V*_0_) of protein hydrolysate solution at different concentrations (0.1%, 0.5%, and 1%) (w/v) was homogenized, using a Moulinex R62 homogenizer, to incorporate air for 1 min at room temperature (25 ± 1°C). The whipped sample was then immediately poured into a 50 mL graduated cylinder, and the total volume was measured (*V*_1_). Foam capacity was expressed as foam expansion after homogenization, which was calculated according to the following equation:

(4) $$\begin{array}{c} \text{FE}\left(\%\right)=\frac{V_{1}-V_{0}}{V_{0}}\times 100. \end{array}$$

Foam stability was calculated as the volume of foam remaining after 30 min at room temperature (*V*_2_). Consider

(5) $$\begin{array}{c} \text{FS}\left(\%\right)=\frac{V_{2}-V_{0}}{V_{0}}\times 100. \end{array}$$

#### 2.7.4. Fat Absorption and Water Holding Capacity

The ability of the CSGHs to absorb fat was determined as described by Shahidi et al. [19] with a slight modification. A 0.5 g of dried CSGH was mixed with 10 mL of corn oil in a 50 mL centrifuge tube. The mixture was kept for 30 min at room temperature (25 ± 1°C) with mixing every 10 min and then centrifuged for 25 min at 2000 g.

The water holding capacity (WHC) of CSGHs was determined according to the method of Okezie et al. [20] with slight modifications. The sample (1 g) was dispersed in 50 mL of distilled water and mixed for 2 min. The mixture was kept at room temperature for 30 min and then centrifuged for 30 min at 5000 g. The two supernatants were filtered with Whatman N°1 filter paper and the volume recovered was measured. The difference between initial volume of distilled water or oil added to the protein sample and the volume of the supernatant was determined, and results were reported as milliliters of water or fat absorbed per gram of CSGHs.

### 2.8. Determination of Antioxidative Activities

#### 2.8.1. DPPH Free Radical-Scavenging Assay

The DPPH free radical-scavenging activity of CSGHs was determined as described by Bersuder et al. [21]. A volume of 500 *μ*L of each sample at different concentrations (0.5 to 5 mg/mL) was mixed with 500 *μ*L of 99.5% ethanol and 125 *μ*L of 0.02 mM DPPH in 99.5% ethanol. The mixtures were then kept for 60 min in dark at room temperature, and the reduction of DPPH radical was measured at 517 nm using a UV-Visible spectrophotometer. The control was conducted in the same condition, except that distilled water was used instead of sample. DPPH radical-scavenging activity was calculated as follows:

(6) DPPH  free  radical-scavenging  activity(%) =(Ac−AhAc)×100,

where *A*_*c*_ is the absorbance of the control reaction and *A*_*h*_ is the absorbance of the hydrolysates. A lower absorbance of the reaction mixture indicated a higher DPPH radical-scavenging activity. Butylated hydroxyanisole (BHA) was used as a standard. The test was carried out in triplicate.

#### 2.8.2. Reducing Power

The sample solution (0.5 mL) at different protein concentrations (0.5 to 5 mg/mL) was mixed with 2.5 mL of 0.2 M phosphate buffer (pH 6.6) and 2.5 mL of 1% potassium ferricyanide. The mixture was incubated at 50°C for 20 min. An aliquot (2.5 mL) of 10% trichloroacetic acid was added to the mixture, followed by centrifugation at 3,000 g for 10 min. The upper layer of solution (2.5 mL) was mixed with 2.5 mL of distilled water and 2.5 mL of 0.1% ferric chloride and the absorbance was read at 700 nm.

#### 2.8.3. DNA Nicking Assay

DNA nicking assay was performed using pCRII TOPO plasmid (invitrogen). A mixture of 10 *μ*L of gelatin hydrolysates at the concentration of 2 mg/mL and plasmid DNA (0.5 *μ*g/well) were incubated for 10 min at room temperature followed by the addition of 10 *μ*L of Fenton's reagent (30 mM H_2_O_2_, 50 *μ*M L-ascorbic acid, and 80 *μ*M FeCl_3_). The mixture was then incubated for 5 min at 37°C. The DNA was analysed on 1% (w/v) agarose gel using ethidium bromide staining.

### 2.9. Determination of Antioxidative Activities in Model Systems

#### 2.9.1. *β*-Carotene-Linoleate Bleaching Model System

The ability of CSGHs to prevent bleaching of *β*-carotene was assessed as described by Koleva et al. [22]. In brief, 0.5 mg *β*-carotene in 1 mL chloroform was mixed with 25 *μ*L of linoleic acid and 200 *μ*L of Tween-40. The chloroform was completely evaporated under vacuum in a rotatory evaporator at 40°C; then, 100 mL of bidistilled water was added, and the resulting mixture was vigorously stirred. The emulsion obtained was freshly prepared before each experiment. Aliquots (2.5 mL) of the *β*-carotene-linoleic acid emulsion were transferred to test tubes containing 0.5 mL of each CSGH (0.5 to 5 mg/mL). The tubes were immediately placed in water bath and incubated at 50°C for 2 h. The absorbance of each sample was then measured at 470 nm. A control consisted of 0.5 mL of distilled water instead of the sample solution. BHA (butylated hydroxyanisole) was used as positive standard. The antioxidant activity of the hydrolysates was evaluated in terms of bleaching of *β*-carotene using the following formula:

(7) $$\begin{array}{c} \text{Inhibition}\left(\%\right)=\left(1-\frac{A_{0s}-A_{120s}}{A_{0c}-A_{120c}}\right)\times 100, \end{array}$$

where *A*_0*s*_ and *A*_0*c*_ are the absorbances measured at initial time of incubation. *A*_120*s*_ and *A*_120*c*_ are the absorbances after 120 min of incubation of the sample and the control, respectively.

#### 2.9.2. Inhibition of Linoleate-Autoxidation Model System

Inhibition activity of* in vitro* lipid peroxidation of CSGHs was determined by assessing their ability to inhibit oxidation of linoleic acid in an emulsified model system [23]. Briefly, freeze-dried gelatin hydrolysates at different concentrations (0.5, 1, 2, 3, 4, and 5 mg/mL) were dissolved in 2.5 mL of 50 mM phosphate buffer (pH = 7.0) and added to 2.5 mL of 50 mM linoleic acid in ethanol (95%). The final volume was then adjusted to 6.25 mL with distilled water.

The obtained mixture was incubated in a 10 mL tube with silicone rubber caps at 45°C for 8 days in dark and the degree of oxidation was evaluated by measuring the ferric thiocyanate values. An aliquot of reaction mixture (0.1 mL) was mixed with 4.7 mL of 75% ethanol followed by the addition of 0.1 mL of 30% ammonium thiocyanate and 0.1 mL of 20 mM ferrous chloride solution in 3.5% HCl. After stirring for 3 min, the degree of color development was measured at 500 nm. *α*-Tocopherol was used as reference and control reaction was conducted without sample. The percentage of oxidation inhibition was expressed as follows:

(8) $$\begin{array}{c} \text{Inhibition}\left(\%\right)=\left(1-\frac{A_{500}\;\text{of}\;\text{sample}}{A_{500}\;\text{of}\;\text{control}}\right)\times 100. \end{array}$$

### 2.10. Stability of Gelatin Hydrolysate

#### 2.10.1. pH Stability

CSGH was dissolved in 10 mL of distilled water at 50 mg/mL of protein concentration; then, the pH was adjusted from 1 to 9 using 1 M HCl or 1 M NaOH and the volume of solution was made up to 25 mL with distilled water. The mixtures were incubated at room temperature (25 ± 2°C) for 1 h. The pH of the mixtures was then adjusted to 7.0 and their volumes were made up to 50 mL with distilled water. The residual antioxidant activities were tested using the *β*-carotene-linoleate bleaching model, DPPH free radical scavenging, and reducing power assays and expressed as the relative activity (%) to those obtained without pH adjustment.

#### 2.10.2. Thermal Stability

CSGH was dissolved in 10 mL of distilled water at a protein concentration of 50 mg/mL; then, pH of gelatin hydrolysate solution was adjusted to 7 and the volume of solution was made up to 50 mL with distilled water. Ten milliliters of the CSGH solution was transferred to screw-capped test tube and placed in a boiling water bath (100°C) for 0, 15, 30, 60, 120, 180, and 240 min. Then, the tubes were immediately cooled in iced water. The residual antioxidant activities were tested using the *β*-carotene-linoleate bleaching model, DPPH free radical scavenging, and reducing power assays and were expressed as relative activity (%) compared to those without heat treatment.

#### 2.10.3. *In Vitro* Gastrointestinal Digestion (GID)

The effect of* in vitro* gastrointestinal digestion of CSGH was evaluated as described by Enari et al. [24] with slight modifications. Briefly, 100 mL of CSGH solution (10 mg/mL) was mixed with 10 mL of phosphate buffer (10 mM, pH = 6.8) and incubated for 2 min at 37°C. Then, 0.5 mL of HCl-KCl buffer (1 M, pH = 1.5) was added to produce an acidic condition, followed by adding 32 U/mL of pepsin solution in 1 M HCl-KCl buffer (pH 1.5) and incubating for 60 min at 37°C (stomach condition). The pH was adjusted to 6.8 with 1 M NaHCO_3_ (1 mL), and the enzyme mixture of bile and pancreatic juice (1 mL) that contained pancreatin (10 mg/mL), trypsin (14,600 U/mL), and bile extract (13.5 mg/mL) in 10 mM phosphate buffer (pH = 8.2) was added to the solution, followed by incubation at 37°C for 3 h to create duodenal condition. To inactivate duodenal enzymes, the test tubes were kept in boiling water for 10 min. The antioxidant activities were tested using the *β*-carotene-linoleate bleaching model, DPPH free radical scavenging, and reducing power assays, during the digestion after 0 (control), 30, 60, 120, 180, and 240 min.

### 2.11. Effect of Gelatin Hydrolysate on Turkey Meat Sausage Lipid Oxidation

Turkey sausage products were formulated using mechanically separated turkey (MST) meat obtained from local processors (Chahia, Sfax, Tunisia). Sausage was prepared as described by Ayadi et al. [25], with slight modification. Dry ingredients such as salt, carrageen, and modified starch were slowly added to the ground MST as powders while processing. Then, cold water was incorporated. The addition of ingredients took less than 5 min at 10°C. The batters were manually stuffed in collagen reconstituted casing and then were heated in a temperature controlled water bath at 90°C until a final internal temperature of 74°C was reached.

After cooling to room temperature, the cooked turkey sausage (placed in polyethylene bag) and the turkey meat sausages were stored at 4°C. The extent of lipid oxidation in each meat sample was determined by the thiobarbituric acid reactive substances (TBARS) assay and the conjugated diene as described by Hogan et al. [26]. The final TBARS value was expressed as mg of malondialdehyde (MDA) equivalents per kg of sample.

### 2.12. Statistical Analysis

Statistical analyses were performed with SPSS version 2.0, professional edition using ANOVA analysis. All results were given as mean value standard deviation of three separate experiments. Differences were considered significant at* P* < 0.05.

## 3. Results and Discussion

### 3.1. Preparation of CSGHs Using Various Proteases

Biological activities of proteins can be increased through hydrolysis with some enzymes, and some peptides or fractions possess stronger activities than others [8]. Thus, gelatin from cuttlefish skin (*Sepia officinalis*), previously extracted [4], was subjected to enzymatic hydrolysis using various proteases: alcalase and crude proteases preparations from* B. mojavensis* A21,* B. subtilis* A26, and* B. licheniformis* NH1.

**Figure 1:**
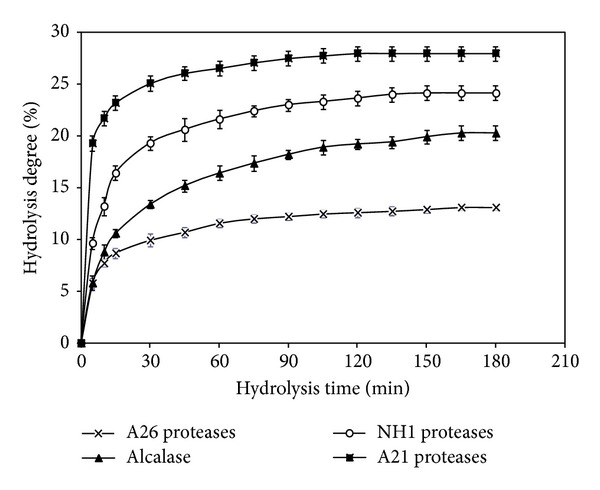
Degree of hydrolysis (DH) of CSGHs during hydrolysis with alcalase, NH1, A21, and A26 proteases at 30 U enzyme/mg substrate. Bars represent standard deviations from triplicate determinations.

After 3 h of hydrolysis, the DHs reached were about 20.3, 26.9, 24.1, and 12.7% with alcalase and crude enzyme preparations from A21, NH1, and A26, respectively. As reported in Figure 1, proteases from* B. mojavensis* A21 showed the highest DH values for cuttlefish gelatin hydrolysis, while crude proteases from* B. subtilis* A26 were the least efficient (*P* < 0.05). The shape of the hydrolysis curves is similar to those previously reported for hydrolysates from muscle of goby [27], sardinelle [28], zebra blenny [29], and gelatin hydrolysates obtained from skin of sole and squid [30].

### 3.2. Chemical and Amino Acid Composition of CSGHs

As shown in Table 1, ash contents ranged between 9.94% and 14.22%; this may be due to the continual addition of NaOH during hydrolysis step. The analysis of mineral content revealed that Na^+^, K^+^, and Cl^−^ were major inorganic matter in CSGHs, while NO_3_^−^, Ca^2+^, Mg^2+^, and SO_4_^2−^ were found at a low level. Sathivel et al. [31] reported that K^+^, Mg^2+^, Na^+^, and Ca^2+^ were abundant in herring and herring by-product hydrolysates and varied with the substrate used.

Gelatin hydrolysates have a high protein content (NH1-CSGH: 74.3%; A21-CSGH: 75.9%; A26-CSGH: 76.3%; and alcalase-CSGH: 78.3%). The high protein content was a result of the solubilization of proteins during hydrolysis, the removal of insoluble undigested nonprotein substances, and partial removal of lipid after hydrolysis [32]. Generally, alkaline proteases exhibited a greater capability to solubilize fish proteins compared to neutral and acidic proteases, with exception of pepsin. Interestingly, all CSGHs had lower levels of lipid compared with salmon protein hydrolysates [32].

The amino acid composition of CSGHs, expressed as residues per 1000 residues, is shown in Table 2. The amino acid composition of CSGHs was similar to the undigested gelatin. The most abundant amino acids were Gly (>32%), Hyp, Pro, Glx, Ala, Asp, and Arg. Generally, the amino acid composition of gelatin hydrolysates is very similar to the parent proteins, being rich in residues of Gly, Ala, Pro, Hyp, Glx, and Asx but poor in Met, Cys, His, and Tyr [8].

The total number of imino acid (Pro and Hyp) residues (between 185 and 194 residues per 1000 residues) was higher than that of collagen from cold-water fish species (16–18%) [33]. Hyp plays a key role in stabilizing the triple stranded collagen helix through the hydrogen bonding ability of its hydroxyl group. Based on total amino acids, essential amino acids made up 12.96%, 13.64%, 13.06%, and 13.33% of A26, NH1, A21, and alcalase-CSGH, respectively. Therefore, they could serve as an excellent source of useful nutrients. Pro and Ala were the most abundant hydrophobic amino acids in all of the gelatin hydrolysates. The alcalase-CSGH had the highest values of Pro, though the ratio with respect to the total hydrophobic amino acid content was steady. Hydrophobic amino acids have been observed in several antioxidant peptide sequences, and Mendis et al. [34] have suggested that the presence of hydrophobic amino acids in the peptide sequences in jumbo squid skin gelatin contributed greatly to its antioxidant properties.

### 3.3. Functional Properties

Functional properties influence the usefulness of an ingredient in food and govern the physical behavior during preparation, processing, and storage.

#### 3.3.1. Solubility

All CSGHs presented typical bell-shaped solubility curves with minimum solubility at pH 5, whereas solubility above 95% was noticeable at the other pH values. However, undigested gelatin showed minimum solubility at pH 6 (data not shown). The solubility increases with the increase of the degree of hydrolysis. At pH = 8.0, the solubility of alcalase-CSGH (DH = 20.26%), NH1-CSGH (DH = 24.12%), A21-CSGH (DH = 26.9%), and A26-CSGH (DH = 12.7%) reached about 97.39, 98, 99, and 96.5%, respectively, significantly higher (*P* < 0.05) than that of CSG (94.5%). It has been suggested that an increase in solubility of protein hydrolysates is due to the reduction of the molecular size and also due to the enzymatic release of smaller polypeptide units from the protein. The smaller peptides are expected to have proportionally more polar residues, with the ability to form hydrogen bonds with water and increase solubility [35].

#### 3.3.2. Emulsifying Properties

Emulsifying activity index (EAI) and emulsion stability index (ESI) of CSG and gelatin hydrolysates at various concentrations (0.1%, 0.5%, and 1%) are shown in Table 3. Results show that the EAI of CSGH was remarkably higher than that obtained with nondigested gelatin (CSG). Best results were obtained by A21 proteases. The increase of EAI using CSGH may be due to lower molecular-weight peptides or partially hydrolyzed gelatin. In addition, EAI values of all CSGH significantly decreased with increasing concentration (*P* < 0.05). Furthermore, the ESI of CSGH and CSG was measured. Results show that the emulsion stability was remarkably high with undigested gelatin. Several authors have described the decrease of emulsifying ability with increasing protein concentration for other fish proteins such as soluble collagen from the skin of sole and squid [30].

Although, in general, a positive relationship between peptide length and emulsifying properties has been reported [5], according to Kristinsson and Rasco [32] there is no clear connection between peptide size and emulsification, suggesting that the physicochemical makeup of the peptides may play an important role in the functional properties. Thus, similar values (240 m^2^/g protein) were found for EAI in both squid and sole hydrolysates at 0.5% (*P* > 0.05). Finally, a concentration of 0.1% of CSGH can be used to possess a higher index of emulsifying activity and emulsifying stability. Protein hydrolysates are surface-active materials and promote an oil-in-water emulsion because of their hydrophilic and hydrophobic groups and their charge [32, 35].

#### 3.3.3. Foaming Properties

Foaming properties are physicochemical characteristics of proteins to form and stabilize foams [36]. Foam expansion (FE) and foam stability (FS) of CSGHs and the control at various concentrations (0.1, 0.5, and 1%) are shown in Table 3.

At the same concentration of hydrolysate used, slight decreases in FE were observed when DH of hydrolysate increased (*P* < 0.05). With the same protein concentration, DH had significant effect (*P* > 0.05) on FE, so the increasing of DH decreases the foaming capacity. At 0.1%, the foaming expansion of alcalase-CSGH (DH = 20.26%), NH1-CSGH (DH = 24.12%), A21-CSGH (DH = 26.9%), and A26-CSGH (DH = 12.7%) reached about 125.1, 126.4, 120, and 131%, respectively. Shahidi et al. [19] reported good foaming properties for capelin protein hydrolysates at low DH (12%). Foam formation is governed by three factors, including transportation, penetration, and reorganization of molecules at the air-water interface [37].

Results show also that the higher foaming stability value was found with A21-CSGHs at different concentrations. The results reveal that when degree of hydrolysis of gelatin increases, the foaming stability decreases. All the CSGHs with a concentration of 1% showed the highest foam stability (Table 3). The stability of foams is a consequence of the well-ordered orientation of the molecules at the interface, where the polar head is located in the aqueous phase and the hydrophobic chain faces the apolar component [38].

#### 3.3.4. Water and Oil-Holding Capacity

Water-holding capacity (WHC) and oil-holding capacity (OHC) are reported in Table 4. OHC and WHC express the quantity of oil and water, respectively, directly bound by the protein and are of great interest, especially in the meat and confectionary industries [35]. As shown in Table 4, A26-CSGH (DH = 12.7%) had significantly higher OHC (4.9 mL oil/g hydrolysate) followed by alcalase-CSGH (DH = 20.26%, 3.1 mL oil/g hydrolysate), NH1-CSGH (DH = 24.12%, 2.6 mL oil/g hydrolysate), and A21-CSGH (DH = 26.9%, 2.4 mL oil/g hydrolysate); this may be attributed to the larger particle sizes in low hydrolyzed proteins.

A decrease in OHC with DH increase has been reported for red salmon head protein hydrolysis [31]. Additionally, WHC increases when the DH increases; for example, A21-CSGH had significantly the highest WHC.

### 3.4. Antioxidant Activity of Cuttlefish Gelatin Hydrolysates

In order to evaluate the antioxidant activity of the cuttlefish gelatin hydrolysates, various antioxidant tests were conducted, including 1,1-diphenyl-2-picrylhydrazyl (DPPH) free radical-scavenging activity, ferric reducing antioxidant power, and inhibition of supercoiled plasmid DNA scission.

**Figure 2:**
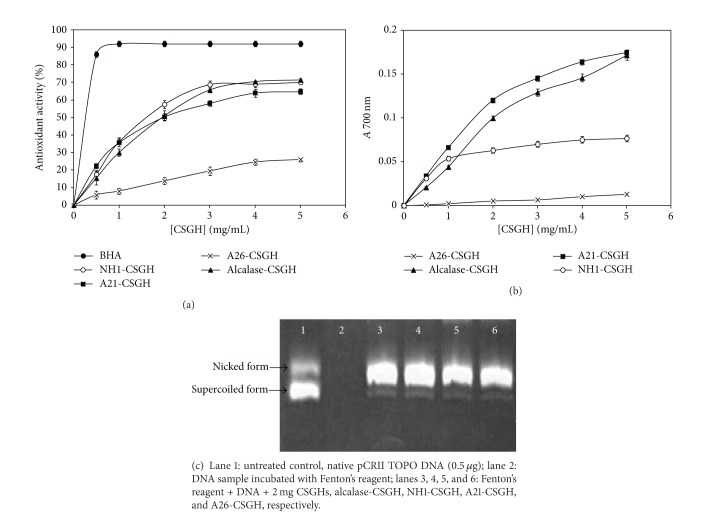
Antioxidant activity using (a) DPPH scavenging, (b) reducing power assay of CSGHs at different concentrations and (c) gel electrophoresis pattern of the plasmid pCRII TOPO incubated with Fenton's reagent in the presence and absence of CSGHs.

Free radical scavenging is a primary mechanism by which antioxidants inhibit oxidative processes. DPPH is a stable free radical that shows maximum absorbance at 517 nm. When DPPH radicals encounter a proton-donating substrate such as an antioxidant, the radicals would be scavenged and the absorbance is reduced [39].  Figure 2(a) shows the results of DPPH radical-scavenging activity of CSGHs at various concentrations. Alcalase-CSGH exhibited the highest antioxidant activity (71% at 5 mg/mL) followed by NH1 and A21-CSGHs with an activity of 69.9% and 64.7%, respectively, while lowest DPPH radical scavenging (26.1%) was obtained with A26-CSGH. However, all hydrolysates showed a lower radical-scavenging activity than BHA at the same concentration.

The results so obtained suggest that the peptides in different hydrolysates, which might be different in terms of chain length and amino acid sequence, contributed to varying degrees of scavenging DPPH radicals. Alcalase and A21 gelatin hydrolysates probably contained more peptides than the other hydrolysates, which are electron donors that could react with free radicals to convert them to more stable products and terminate the radical chain reaction.

Ferric reducing antioxidant power (FRAP) generally measures the reducing ability against ferric ion (Fe^3+^). This ability indicates the ability of hydrolysates to donate electron to the free radical [9]. As shown in Figure 2(b), the reducing power activities of the different gelatin hydrolysates are concentration dependent. Alcalase-CSGH (DH = 20.26%) and A21-CSGH (DH = 26.9%) have, respectively, a ferric reducing antioxidant power three- and ninefold higher (*P* < 0.05) than that of A26-CSGH (DH = 12.7% at 5 mg/mL). Increases in reducing power of hydrolysate with increasing DH have been reported in blacktip shark (*Carcharhinus limbatus*) skin gelatin hydrolysate prepared using papaya latex enzyme [3], loach (*Chromobotia macracanthus*) protein hydrolysates [40], and gelatin hydrolysate from bigeye snapper (*Lutjanus lutjanus*) during a simulated gastrointestinal digestion [36].

The chemical activity of the hydroxyl radical is the strongest among reactive oxygen species (ROS). It easily reacts with biomolecules, such as amino acids, proteins, and DNA [41]. Therefore, scavenging of the hydroxyl radical is probably one of the most effective defenses of a living body against various diseases.

The hydroxyl radical-scavenging abilities of CSGHs using DNA nicking assay are shown in Figure 2(c). Lane 1 represents the untreated plasmid (native DNA) with its two forms: the upper one is open-circular (nicked) DNA and the faster migrating band is supercoiled (closed circular) plasmid. The incubation of plasmid DNA with Fenton's reagent in the absence of CSGH resulted in the disappearance of both forms, indicating that DNA was completely degraded (lane 2). Interestingly, all gelatin hydrolysates exhibited moderate protection against hydroxyl radical induced DNA breakage (Figure 2(c), lane 3 to lane 6).

### 3.5. Determination of Antioxidative Activities in Model Systems

#### 3.5.1. *β*-Carotene-Linoleate Bleaching Model System

**Figure 3:**
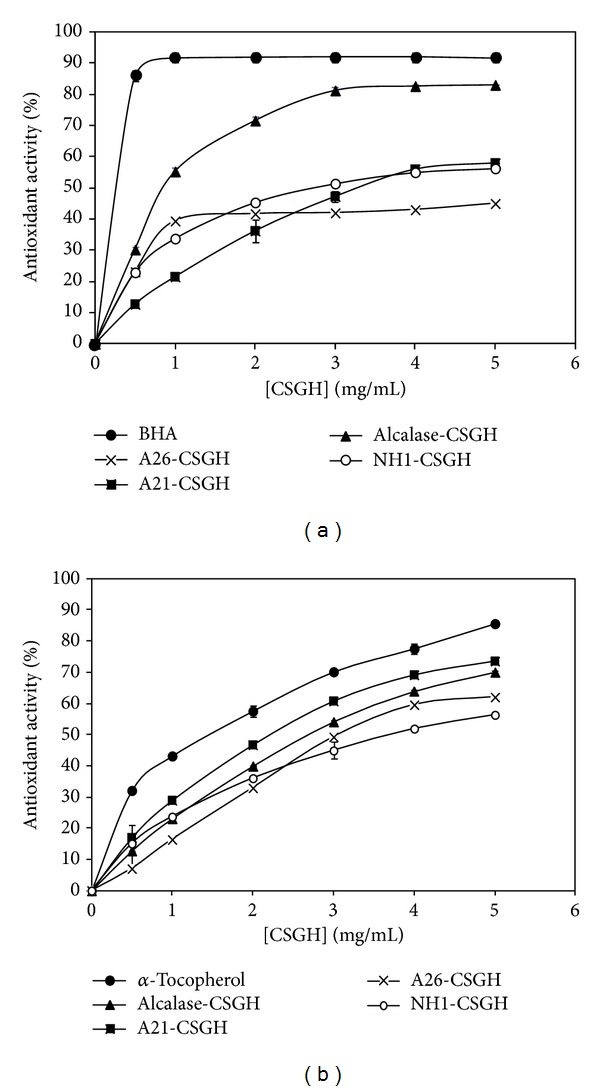
(a) *β*-Carotene bleaching method and (b) inhibition of lipid peroxidation of CSGHs at different concentrations.

The antioxidant assay using the discoloration of *β*-carotene is widely used to measure the antioxidant activity of bioactive compounds, because *β*-carotene is extremely susceptible to free radical-mediated oxidation of linoleic acid [42]. The presence of antioxidant in linoleic acid emulsion system hinders *β*-carotene bleaching, due to the chain-breaking inhibition of lipid peroxidation by neutralizing the linoleic free radical formed. The antioxidant activities of CSGHs as measured by *β*-carotene bleaching are shown in Figure 3(a). All hydrolysates prevent *β*-carotene bleaching by donating hydrogen atoms to peroxyl radicals of linoleic acid. As can be seen, the antioxidant activity of CSGHs increased with increasing sample concentration. Alcalase-CSGH which had the lowest reducing power and DPPH radical-scavenging activity showed the highest ability to prevent *β*-carotene bleaching with 82.1% inhibition at 5 mg/mL and the hydrolysate prepared by NH1 proteases showed the lowest. However, the inhibition of *β*-carotene bleaching by all hydrolysates was lower than that obtained with BHA (92%).

#### 3.5.2. Inhibition of Linoleic Acid Antioxidant Activity

*In vitro* lipid peroxidation inhibition activities of CSGHs were determined by assessing their ability to inhibit oxidation of linoleic acid in an emulsified model system.

All hydrolysates could act as significant retarders (*P* < 0.05) of lipid peroxidation and activity increased with increasing concentrations. The comparative study between CSGHs and commercial antioxidant (*α*-tocopherol) on the inhibition of lipid peroxidation was conducted and illustrated in Figure 3(b). The autoxidation of CSGHs was slightly lower than that of *α*-tocopherol. This indicates that CSGHs had an effective capacity to inhibit lipid peroxidation. A21-SCGH exhibited the highest inhibition activity (72.1% ± 0.57) followed by alcalase-CSGH (71.5% ± 1.4) at a concentration of 5 mg/mL.

### 3.6. Stability of Selected Gelatin Hydrolysate and Application

Cuttlefish skin gelatin hydrolysates prepared using alcalase which showed the highest *β*-carotene bleaching, radical scavenging, lipid peroxidation inhibition, and reducing power activity were selected for stability studies.

#### 3.6.1. pH and Thermal Stability

**Figure 4:**
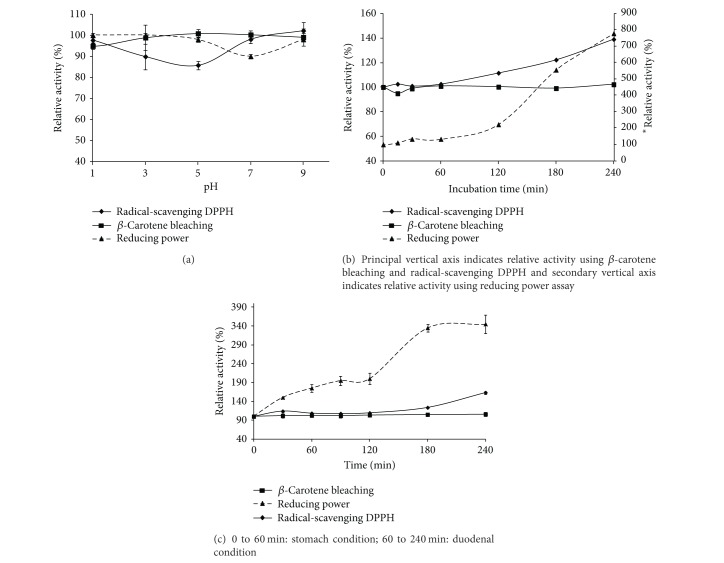
pH (a), thermal (b), and digestive (c) stabilities of alcalase-CSGH as monitored by *β*-carotene bleaching, radical-scavenging DPPH, and reducing power assay. Bars represent standard deviation (*n* = 3).

The relative antioxidant activity (*β*-carotene-linoleate bleaching model, DPPH free radical scavenging, and reducing power assays) of the CSGH obtained by alcalase proteases at different pH (1 to 9) is presented in Figure 4(a). The antioxidant activity of alcalase-CSGH tested using *β*-carotene-linoleate bleaching model and DPPH free radical scavenging was stable over the pH range of 1–9 (*P* > 0.05). At pH 7, the ferric reducing activity decreased slightly, compared with that without pH adjustment. Kittiphattanabawon et al. [3] proved that the chelating activity of gelatin hydrolysate increased by 800% after pH adjustment, possibly due to the changes of charges in peptides.

The effect of thermal treatment (0 to 240 min, at 100°C) on antioxidant activity of alcalase-CSGH is shown in Figure 4(b). Radical-scavenging and reducing power activities increased by approximately 140% and 780%, respectively, after 240 min of heating treatment (*P* < 0.05). You et al. [40] also found that heat treatment was beneficial for increasing the antioxidant activity of peanut antioxidant hydrolysate. Thus, it suggests that alcalase-CSGH had a potential for application in any food system over pH range of 1–9 and thermal processed at 100°C for up to 240 min without loss or with increasing of activity.

#### 3.6.2. *In Vitro* Gastrointestinal Digestion

*In vitro* gastrointestinal enzyme incubation provided an easy process to imitate the fate of this hydrolysate under oral administration. The relative antioxidant activity alcalase-CSGH, as monitored by *β*-carotene-linoleate bleaching model, DPPH free radical scavenging, and reducing power assays, after different digestion times is presented in Figure 4(c). No change in *β*-carotene-linoleate bleaching capacity was observed at any digestion times (0–240 min) (*P* > 0.05). It was observed that radical-scavenging activity increased by 163% during the last 15 min of gastrointestinal digestion (*P* < 0.05). Reducing power also increased by 175% during the first hour (stomach condition); then, it increased by 345% at the end of the GID (time = 240 min) (*P* < 0.05). It was observed that antioxidative peptides were modified by enzyme digestion to enhance their radical-scavenging and reducing power activities.

Our results are in accordance with those obtained by You et al. [40] who have proved that digestion of papain-hydrolysed loach peptide with pepsin and pancreatin increases the antioxidant activities. In addition, Nalinanon et al. [43] demonstrated that antioxidant peptides were most likely stable in real digestion system after ingestion in both stomach and intestine, which have high proteolytic activity under acidic and alkaline pH, respectively.

#### 3.6.3. Prevention of Lipid Peroxidation in Meat Sausage System

**Figure 5:**
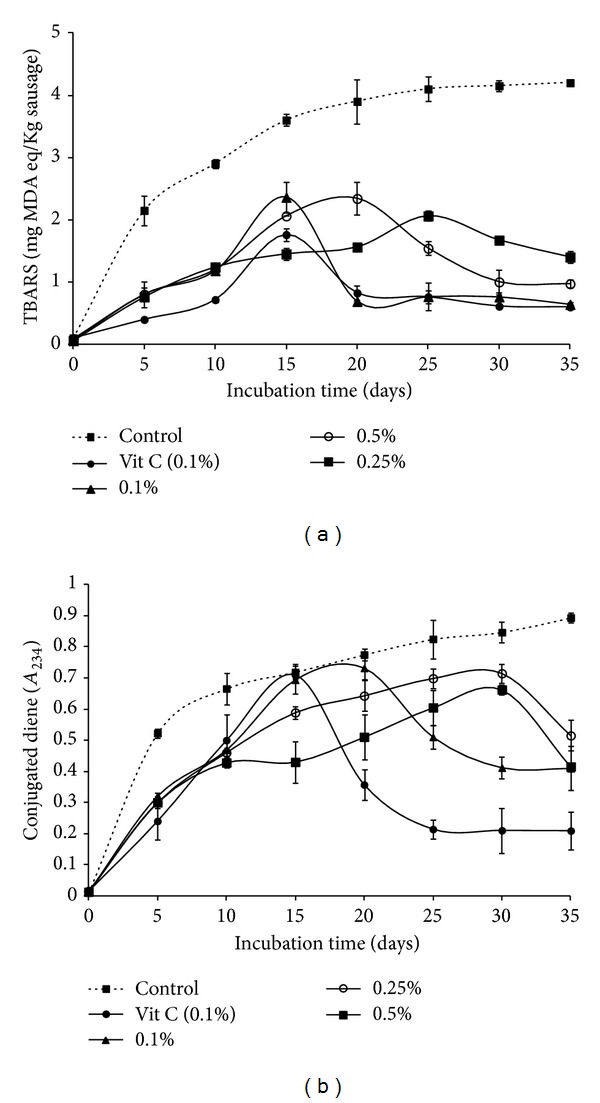
Lipid oxidation of meat sausage added with alcalase-CSGHs at different levels: (a) thiobarbituric acid reactive substances (TBARS) and (b) conjugated dienes. Bars represent the SD from triplicate determinations.

Protein and gelatin hydrolysates have been shown to effectively inhibit lipid peroxidation in meat products [44], suggesting that food proteins could be utilized to develop specific hydrolysates as natural antioxidants for improving shelf-life of lipid-rich food products. In this study, lipid oxidation in meat sausage containing alcalase-CSGH at levels of 0 (Control), 0.1, 0.25, and 0.5 (g per 100 g of sausage) and vitamin C (0.1%) was monitored during storage at 4°C for 35 days, using TBARS and conjugated diene assays (Figure 5).

Generally, the TBARS of cooked turkey meat sausage increased during storage period and reached the maximum after 15-day storage except for concentrations 0.25% and 0.50% which reached their maximum after 20 and 25 storage days, respectively (Figure 5(a)). Thereafter, the decrease in TBARS was observed until the end of storage (*P* < 0.05). This was probably due to the loss of oxidation products formed, particularly low MW volatile compounds. Malondialdehyde and other short-chain products of lipid oxidation are not stable and are decomposed to alcohols and acids, which are not determined by the TBARS test [45].

The concentration of conjugated dienes significantly increased in all samples, followed by a decrease (Figure 5(b)). The rate of increase varied with the samples and concentrations used. The formation of conjugated dienes occurs at the early stages of lipid oxidation and hydroperoxides are expected to decompose to create secondary products [45]. The decrease or reaching of a stagnant level in conjugated dienes was generally accompanied by an increase in TBARS [46].

All gelatin hydrolysates could inhibit the early stages of lipid oxidation (formation of conjugated dienes or hydroperoxides) as well as retard propagation of the oxidation process (degradation of hydroperoxide to TBARS) [37]. A concentration of 0.50% of CSGH was generally more effective in inhibiting the lipid oxidation in the meat sausage system than other concentrations of CSGH and vitamin C as shown by the lower conjugated diene formation throughout the incubation. Additionally, an increase in TBARS values was lower than the control and the efficiency in retarding lipid oxidation was concentration dependent.

## 4. Conclusion

The objective of this work was to investigate some functional properties and the potential antioxidant effect of CSGHs prepared with different microbial enzyme preparations. Cuttlefish skin gelatin hydrolysates obtained with different alkaline proteases resulted in a product with an excellent solubility over a wide pH range. In addition, CSGHs prepared by treatment with different enzyme preparation, which displayed different spectra of substrate specificity, exhibited, to a variable extent, antioxidant activity in various* in vitro* antioxidant systems. The overall antioxidant action of CSGHs is likely attributed to the cooperative effects of several mechanisms, and the differences between functional properties and biological activities of the hydrolysates are in particular due to diversity of peptides, which might be different in terms of chain length and amino acid sequence. From the results, cuttlefish skin gelatin hydrolysates have also a high nutritional value, based on their amino acid profile. Therefore, alcalase-CSGH exhibits a good antioxidant activity in turkey meat sausage model and can be a promising natural substrate that could be utilized in different food systems.

## Figures and Tables

**Table 1:** Proximate composition (g/100 g dry matter) and mineral content (*μ*g/g) of CSGHs.

	Alcalase-CSGH	NH1-CSGH	A26-CSGH	A21-CSGH
Moisture (%)	9.62 ± 0.002^b^	8.34 ± 0.32^c^	10.32 ± 0.076^a^	7.92 ± 0.81^d^
Ash (%)	10.22 ± 0.003^c^	13.87 ± 0.015^b^	9.94 ± 0.01^c^	14.22 ± 0.026^a^
Fat (%)	0.35 ± 0.01^c^	0.72 ± 0.02^a^	0.50 ± 0.03^b^	0.45 ± 0.01^b^
Protein (%)	78.34 ± 0.12	74.29 ± 0.3	76.33 ± 0.27	75.94 ± 0.22
Mineral content (*μ*g/g)				
Ca^2+^	74^d^	84^b^	96^a^	77.1^c^
Na^+^	360^b^	384.4^a^	334.5^c^	390.7^a^
K^+^	827^d^	1100.2^a^	990^c^	1021^b^
Mg^2+^	104^a^	95^b^	101^a^	95^b^
Cl^−^	168^b^	170^c^	169.1^b^	190.1^a^
NO_3_^−^	25.5^d^	33.6^b^	34.4^a^	32.4^c^
SO_4_^2−^	36.5^a^	24^d^	31.5^b^	29.3^c^

^a,b^Different letters in the same line indicate significant differences (*P* ≤ 0.05).

**Table 2:** Amino acid composition of CSGHs (number of residues/1000 residues).

Amino acids	CSG	A26-CSGH	NH1-CSGH	A21-CSGH	Alcalase-CSGH
Asx^a^	63	62	61	62.5	60
Thr^b^	23	22	24.6	22.4	22
Ser	49	45	44	42.3	40.7
Glx^a^	92	98	96.4	97.4	89.4
Gly	321	320	316	317	318
Ala	81	87.3	85.4	98.6	97.4
Val^b^	22	12	13.6	13.5	12.1
Met^b^	6	6.3	6.4	6.9	7.1
Ile^b^	22	22.6	23.4	22.1	23.1
Leu^b^	29	19.7	20.4	18.7	20
Try	5	5.9	6.2	6.1	5.8
Phe^b^	10	9.6	9.9	9.3	9.4
His^b^	18	15	14	14	17
Lys^b^	13	32	34	33	32
Arg	51	53.6	52.7	51.2	52
Cys	0	0	0	0	0
Pro	96	92	94	90	98
Hyp	84	97	98	95	96
TAA^c^	1000	1000	1000	1000	1000
THAA^c^	587.0	569.5	569.1	576.1	585.1
TEAA/TAA (%)^c^	14.3	13.92	14.63	13.99	14.27

^a^The aspartic and glutamic acid contents include, respectively, asparagines and glutamine, Asx = Asp + Asn; Glx = Glu + Gln.

^b^Essential amino acids.

^c^TAA = total amino acids; THAA = total hydrophobic amino acids; TEAA = total essential amino acids.

**Table 3:** Emulsion activity index (EAI), emulsion stability index (ESI), foam expansion (FE), and foam stability (FS) of cuttlefish skin gelatin hydrolysates at various concentrations.

	Concentration % (g/100 mL)	EAI (m^2^/g)	ESI (min)	FE (%)	FS (%)
CSG	0.1	15.21 ± 0.2^dC^	53.29 ± 0.8^aA^	100.23 ± 0.9^dC^	49.6 ± 1.2^dC^
	0.5	17.22 ± 0.2^dB^	51.28 ± 0.1^aB^	103.44 ± 0.5^eB^	83.1 ± 10.0^aB^
	1	23.67 ± 0.3^aA^	49.14 ± 0.8^aB^	113.7 ± 1.53^dA^	105.3 ± 0.32^aA^

Alcalase-CSGH	0.1	58.21 ± 1.2^bA^	25.21 ± 0.9^cA^	125.1 ± 5.1^bB^	65.1 ± 5.5^bB^
	0.5	33.12 ± 0.8^aB^	18.95 ± 0.75^bcB^	125 ± 4.7^cB^	68 ± 4.9^cB^
	1	10.24 ± 0.2^bC^	14.01 ± 0.7^dC^	129.2 ± 3.1^bA^	84 ± 4.8^bA^

NH1-CSGH	0.1	48.01 ± 2.1^cA^	18.14 ± 1.6^dA^	126.4 ± 4.7^bB^	60.3 ± 3.7^cC^
	0.5	13.21 ± 0.9^eB^	17.01 ± 1.1^cB^	128 ± 3.5^bAB^	67 ± 4.2^cB^
	1	5.47 ± 0.2^cC^	19.51 ± 0.9^bA^	130 ± 4.0^bA^	74 ± 5.9^cA^

A21-CSGH	0.1	68.24 ± 1.2^aA^	16.36 ± 2.3^eA^	120 ± 2.6^cB^	69.7 ± 1.4^aC^
	0.5	22.25 ± 1.7^cB^	14.7 ± 1.9^dB^	122 ± 3.1^dAB^	74.4 ± 1.9^bB^
	1	6.26 ± 0.33^cC^	13.9 ± 2.0^dB^	125 ± 2.5^cA^	88.1 ± 2.7^bA^

A26-CSGH	0.1	50.76 ± 4.0^cA^	33.21 ± 1.7^bA^	131 ± 2.3^aB^	47.6 ± 0.9^eC^
	0.5	31.69 ± 2.4^aB^	19.46 ± 1.9^bB^	133 ± 3.1^aAB^	51 ± 1.5^dB^
	1	9.87 ± 0.7^bC^	15.94 ± 0.4^cC^	134.8 ± 4.2^aA^	59 ± 2.3^dA^

Values are given as mean ± SD from triplicate determinations.

Different letters in the same column within the same concentration indicate significant differences (*P* < 0.05).

Different capital letters in the same column within the same hydrolysate sample indicate significant differences (*P* < 0.05).

**Table 4:** Water and oil-holding capacity of CSGHs.

	WHC (mL/g)	OHC (mL/g)
CSG	2.15 ± 0.72^d^	3.52 ± 0.28^b^
A26-CSGH	1.9 ± 0.1^d^	4.9 ± 0.1^a^
Alcalase-CSGH	2.81 ± 0.2^c^	3.2 ± 0.1^c^
NH1-CSGH	3.51 ± 0.1^b^	2.6 ± 0.2^d^
A21-CSGH	3.9 ± 0.2^a^	1.7 ± 0.1^e^

WHC = water-holding capacity (mL of water absorbed/g of sample); OHC = oil-holding capacity (mL of oil absorbed/g of sample); values are given as mean ± SD from triplicate determinations. ^a,b^Different letters indicate significant differences (*P* ≤ 0.05).
